# Lexical Processes and Eye Movements in Neglect Dyslexia

**DOI:** 10.1155/2002/789013

**Published:** 2002-10-01

**Authors:** Giuseppe di Pellegrino, Elisabetta Làdavas, Claudio Galletti

**Affiliations:** ^1^ School of Psychology University of Wales Bangor Wales UK; ^2^ Department of Psychology University of Bologna Bologna Italy; ^3^ Institute of Physiology University of Bologna Bologna Italy

**Keywords:** neglect dyslexia, eye movement, lexical access, spatial attention, reading

## Abstract

Neglect dyslexia is a disturbance in the allocation of spatial attention over a letter string following unilateral brain damage. Patients with this condition may fail to read letters on the contralesional side of an orthographic string. In some of these cases, reading is better with words than with non-words. This word superiority effect has received a variety of explanations that differ, among other things, with regard to the spatial distribution of attention across the letter string during reading. The primary goal of the present study was to explore the interaction between attention and lexical processes by recording eye movements in a patient (F.C.) with severe left neglect dyslexia who was required to read isolated word and non-word stimuli of various length.

F.C.’s ocular exploration of orthographic stimuli was highly sensitive to the lexical status of the letter string. We found that: (1) the location to which F.C. directed his initial saccade (obtained approximately 230 ms post-stimulus onset) differed between word and non-word stimuli; (2) the patient spent a greater amount of time fixating the contralesional side of word than non-word strings. Moreover, we also found that F.C. failed to identify the left letters of a string despite having fixated them, thus showing a clear dissociation between eye movement responses and conscious access to orthographic stimuli.

Our data suggest the existence of multiple interactions between lexical, attentional and eye movement systems that occur from very initial stages of visual word recognition.

## 1. Introduction

Visual neglect following unilateral brain lesion can sometimes result in a reading impairment known as neglect dyslexia [50]. People with this condition tend to concentrate their errors on the side of a word or sentence that is contralateral to their lesioned hemisphere. For example, after a right hemisphere lesion, patients may misread the initial (left-most) letters of single words, producing substitution (e.g., boat → "coat"), insertion (e.g., love → "glove"), or omission (e.g., cage → "age") errors. Manifestations of neglect dyslexia may vary greatly from one patient to another. For example, whereas several cases have shown that neglect dyslexia and generalized visual-spatial neglect co-occur, there are observations of neglect dyslexia in the absence of neglect for non verbal material [2,43, 51]. Also, some patients are impaired in reading isolated words and text, whereas others produce errors in words, while text reading remains intact [7,43,51]. Similarly, some neglect patients identify better the left half of word strings than non-word strings [1,4,6,28,31, 32,51], whereas there are instances in which this does not occur (see Ellis et al. [13]). Despite this clinical heterogeneity, most authors agree that neglect dyslexia is to be attributed to some deficit in the allocation of spatial attention to the lesioned side of space [1,4,13, 28,31,32,51]. On this perspective, contralesional visual stimuli would gain a poorer perceptual representation than ipsilesional items following a brain damage, with

ISSN 0953-4180/01/02/$8.00 © 2001/2002 – IOS Press. All rights reserved the result that attentional selection is biased towards information present in the intact region of space.

The present paper reports a detailed examination of the eye movement made by a patient with neglect dyslexia, F.C., as he inspected word and non-word strings for verbal report.

F.C. is of particular interest because his reading errors tend to be more frequent for words than non-words; namely he demonstrates a word superiority effect. The primary goal of the current paper is therefore to explore further the relationship between attentional deficit in neglect dyslexia and the relative sparing of word versus non-word reading. Although this issue has provoked a large amount of research (see Behrmann [3], Ellis et al. [14], and Riddoch [51] for extensive reviews), its interpretation remains somewhat controversial. Specifically, one explanation of such word superiority effect assumes that neglect dyslexics are using letters in the non neglected (ipsilesional) portion of a word to infer, or guess, the identity of letters in the neglected portion, due to the constraints of the orthographic lexicon [43]. One clear prediction that follows from this account is that stimulus lexicality should not affect the allocation of attention across word and non-word strings, even though a performance advantage may still be found for words due to the guessing process.

Another class of account for the word superiority effect in neglect dyslexia rests on the assumption that partially processed letters on the contralesional side receive top-down support from lexical representations stored in memory [1,4,6,56]. This higher order lexical knowledge may enhance and interpret the impoverished perceptual inputs, thus improving the reading performance and giving rise to a word superiority effect. Of course, this type of reasoning does not apply to non-word strings which do not have prior representations in the brain. A similar top-down account has been proposed by Brunn and Farah [6], who have further postulated that lexical access may feed back on an early attention mechanism which triggers a reallocation of attention to the left to encompass the area subtended by the word. This latter explanation also implies that the lexical status of the stimulus (word /non-word) must be recognised very early during the word naming process, possibly prior to the accurate identification of the target in order to trigger a reorienting of attention to the left side of the stimulus. This view appears in line with a recent study of Làdavas and colleagues [31,32] who used an experimental procedure that directly tapped the lexical (and semantic) information of words incorrectly read aloud by the patients. Làdavas et al. [31,32] showed that neglect patients performed normally on a lexical decision task despite of being unable to read the letter strings, producing always non-word responses. Furthermore, data from a recent event related potential (ERP) study of word recognition are highly consistent with the notion that lexical codes are activated early during reading [54].

To adjudicate between these contrasting hypotheses we examined directly the allocation of attention over isolated word and non-word strings while F.C. was asked to read those strings. To assess the allocation of spatial attention, we analysed the patterns of eye movements during reading. We chose this technique because work in normals and brain damaged patients has shown that a tight link (or even identity, according to one proposal [53]) exists between mechanisms that control attention and those that drive eye movements [9,16,18, 26,27,49,52,59,61]. The attention-eye movement relationship appears particularly strong in complex information processing tasks such as reading [47]. Furthermore, eye movement analysis allows for the possibility of studying moment-to-moment cognitive processes during reading of words and non-words, so that an effect of lexical activation may be revealed at an early stage of word processing [35,42,47]. In the present study two oculomotor measures were used: the initial landing position in a letter string, and the spatial distribution of fixation time as a function of letter position in words and non-words. The first landing location was used to assess early cognitive processing of the target string, particularly to examine whether the lexical status of a letter string influences very early the allocation of attention during reading. The fixation time served as a global index of the distribution of attention during word and non-words reading. If the relative sparing of words occurs without a contribution of spatial attention, as the “guessing” hypothesis holds, then there should be no difference in the distribution of eye movements across word and non-word strings. If, however, the better performance with words is due to the reallocation of attention to the left, then we should find a greater amount of time spent fixating the left side of words than non-words.

Another focus of interest of this paper concerns the relationship between neglect dyslexia and disturbance of eye movement behaviour. Deficits of oculomotor pattern have been reported previously in neglect patients [5,16,17,22,58]. For instance, a number of studies have shown that neglect patients have a pronounced tendency to scan and fixate only the ipsilesional side of space, are slower at initiating eye movements toward contralesional stimuli, and begin to explore a visual scene from the right (rather than left) of the midline. However, very few studies have investigated the patterns of eye movements made by patients with neglect dyslexia during reading [24,40]. By examining in fine detail the distribution of eye movement made by F.C. as he viewed letter strings, we investigated the possibility that the failure in reporting letters at initial locations of strings may reflect a primary deficit in making leftward saccades to those locations. Moreover, if F.C.'s neglect dyslexia arises from faulty eye movement programming and/or execution then we would also expect no difference in the oculomotor pattern during the reading of words and non-words.

Finally, recent eye movement studies have reported that in some instances neglect patients fail to acknowledge the presence of stimuli on the left even after prolonged scan and fixation of those stimuli [33,59]. Accordingly, we also examined whether F.C. failed to identify the left letters of a string despite having fixated them. Such a result would strongly endorse the view that neglect (dyslexia) is an high-order disorder of spatial awareness that cannot solely be attributed to a primary perceptual or motor impairment.

## 2. Methods

### *2.1. Subjects*

1) F.C., a 63-year-old retired right-handed man, was admitted to the "Fraticini" hospital, Florence, in August 1997. He was a native speaker of Italian and had completed 5 yrs of formal schooling. The initial neurological examination revealed a spastic hemiparesis of his left arm and leg, as well as a left-sided hemi-anaesthesia. A CT scan showed a large hypodense area in the right fronto-temporo-parietal region, consistent with an ischemic infarction in the territory supplied by the right middle cerebral artery. At the time of testing, which was initiated 2 months after the onset of his stroke, F.C. was still wheelchair-bound and showed significative left sensory loss. The patient did not exhibit deficit of visual acuity, as demonstrated by clinical examination. Goldman perimetry showed that F.C. had an homonymous left visual field defect. However, in this patient, approximately the central 20 degrees of the left visual field were spared. F.C. did not show any signs of speech or verbal comprehension deficit, and appeared to be alert, cooperative, and well oriented with regard to time and place. Severe unilateral neglect was diagnosed on the basis of a number of standard pencil-and paper tests, including line bisection, where F.C. cut the line to the right of centre, as well as "bell" and "H" cancellation tests, in which he missed items on the left side of the page. In addition, neglect symptoms were observed during reading a short passage of text as well as single words.
2) A control group of subjects with no history of neurological disease was made up of 4 subjects (1 men and 3 women). These subjects were selected to approximately match the patient's age and years of education. Control subjects were all Italian speaking and had normal uncorrected vision.

### *2.2. Apparatus*

Subjects were seated in a chair in a dimly illuminated room with their head stabilised straight-ahead by means of an adjustable forehead and chin rest. Head movements were further restrained by a strap which passed behind the head. The stimuli for the experiment were generated by an IBM-compatible Pentium Plus computer using custom software and displayed on a colour monitor ($24 \times 32$ cm). The video screen was centred on the midsaggital plane of the subject's head and was viewed binocularly from a distance of approximately 40 cm. Horizontal and vertical eye movements were monitored using an infrared corneal reflection oculometer (BOUIS INSTRUMENTS, Germany) positioned in front of the subject's left eye. The eye-movement tracker had a high resolution (about 5 minutes of arc), and its output was linearly related to eye position within an area of approximately plus or minus 10 degrees of visual angle (both horizontally and vertically). The analogue eye movement signals were sampled every 2 ms (i.e., 500 samples per second), digitised by a labdriver interface, and stored on a hard-disk for off-line analysis.

### *2.3. Stimulus material*

Stimuli comprised 56 letter strings, 14 each of 6, 9, 10 and 11 letters in length. Each string was always composed of upper-case letters ($0.6 \times 0.7$ cm) separated by a single character space ($0.6 \times 0.7$ cm). Thus, a 9 letter string occupied a total of 17 character spaces ($10.2 \times 0.7$ cm). Stimuli were printed in white against a black background, and were displayed horizontally at the center of the video screen, one at a time. Half of the stimuli ($n = 28$) were common Italian words, and the remaining half ($n = 28$) were non-words generated by changing two letters of each word. The substituted letters were located at the beginning and at the end of the stimulus. All non-words strings were pronounceable and orthographically legal. All the word strings were concrete nouns and denoted either living or non-living items. Compounds words were not used. Word and non-word stimuli were pseudorandomly divided into two lists of 28 items each. Subject received the two lists in separate blocks of trials. Within each block, order of string presentation was fixed and the same for all subjects.

### *2.4. Procedure*

*Calibration task.* Before collecting the data on the reading task, the eye-position signals were calibrated. To this end, the subject viewed a central fixation cross (0.5 cm) and four outline squares (sides of approximately 0.5 cm), located at $10^{a}$ to the right, left, top and bottom of the cross. First, the zero point calibration was established by having the subject to gaze at the central cross. Then, he was asked to fixate on the centre of each of the 4 squares in turn. The order of square fixation was randomised to avoid any possible cueing effects. As F.C. had neglect, care was taken to ensure that he located each square with his eyes (particularly the left-sided square). To this purpose, the subject was required to track a pen that was moved slowly from the central cross to each calibration square in random order. The calibration routine was repeated before each trial to reduce any inaccuracy in eye-position coordinates produced by small head movement during reading aloud the string. Once the coordinates were established, the trial was commenced.

*Reading task.* At the start of each trial a fixation cross was presented in the centre of the video screen. Following a 1 s period of steady central fixation, the experimenter gave a ready signal, and then pressed a button to initiate the display. The central cross was extinguished and, after 100 ms, the stimulus was displayed for 4800 ms. Subjects were told to read the string normally, and to report verbally what they had read. If subjects named the target string before the presentation time had elapsed, the experimenter pressed the space bar of the keyboard to blank the screen. The subject's responses were recorded by the experimenter during the session. Before the experiment, subjects were informed that some of the strings would not be words, and read few practice word and non-word strings to become familiar with the stimuli. During stimulus presentation there was no restraint on eye movements. Eye-position recording started 100 ms before stimulus presentation and continued until the string went off. The subject was requested to stay as still as possible and to try not to blink during the recording period. A short block of practice trials was given to ensure that subjects could understand and comply with the instructions. A short rest occurred halfway through the experiment, but a longer break was allowed if subjects appeared fatigued.

### *2.5. Analysis*

For off-line analysis, the eye-movements records were automatically plotted onto the video screen as a scan path, and superimposed on the original stimulus. Each trial was examined individually. The region of space occupied by each string was divided into a number of equally wide horizontal sectors, one for each letter composing the string (e.g., 9 sectors for 9 letter string). Sectors were numbered from the centre of the string to outwards, with right-sided sectors coded as positive, and left-sided sectors as negative. In odd-numbered strings, the middle letter in the string was encoded as 0. The eye-movement data were analysed in terms of first landing location and fixation time. For first landing location, we calculated direction and amplitude of the initial saccade following stimulus onset. Landing location was expressed in terms of letter position in a target string (rather than in degree), so that $-1$ means that the reader landed one letter to the left of the string center. We defined a saccade as any eye movement which had a velocity greater than $30^{\circ}$/sec (i.e., clearly distinguishable from drift). We also measured the latency of the first saccade as the time from the onset of the stimulus to the point at which the start of the saccade was detected. For measures of fixation time, we used the amount of time subjects spent fixating on each letter of a string. Note that total fixation time (which is the sum of all fixations on a letter string) could not exceed stimulus duration (4800 ms).

## 3. Results

### *3.1. Reading performance*

Reading errors (i.e., omitting or misreading one or more letter in a string), made in response to word and

Table 1
Percent accuracy for word and non-word strings of different lengths for F.C. and control subjects

| String length in letters | F.C. | | Control subjects | |
|---|---|---|---|---|
| | W | N-W | W | N-W |
| 6 | 57.1 | 14.3 | 100 | 91.5 |
| 9 | 42.9 | 0 | 100 | 100 |
| 10 | 14.3 | 0 | 100 | 82.5 |
| 11 | 14.3 | 0 | 100 | 87 |

non-word targets, were classified as "neglect", or "visual" errors, depending on whether they involved the left half of the orthographic stimulus, or some other position. Table 1 shows the percentage of correct responses as a function of lexicality and stimulus length, separately for F.C. and control subjects.

Control subjects performed at ceiling with words and produced relatively few errors in response to non-word stimuli (10%). All errors were of the visual type, and included letter omissions, additions, or substitutions, usually involving letters located in the middle of the string. Percentage of errors did not depend on the length of the string.

In striking contrast, all F.C.' errors were of the neglect type in which the right side of the target and the response are identical but no common letters appear to the left of a 'neglect point' (following Ellis et al. [13]). Errors were significantly fewer with words (68%) than with non-words (96%) stimuli. There was a clear effect of string length, with reading accuracy decreasing as the number of letters increased (see Table 1). As to the lexicality of the responses, the majority of F.C.'s errors were non-words (77%) which, however, occurred more frequently in response to non-word targets (96%) than word targets (58%). Overall, the dominant neglect error in F.C. was that of letter omission, which occurred on 84% and 96% of errors made in response to word and non-word targets, respectively. The remaining neglect errors consisted nearly always of letter substitutions. F.C. never attached additional letters to the left of the string, but occasionally he inserted a letter in the left side of the target (1% of errors). We also looked more closely at the distribution of errors as a function of letter position in the string in the corpus of words and non-words. Each letter of the stimulus string was scored as to whether it was reported correctly or not. If the letter did not appear in the response, or was substituted with another letter, 1 error was recorded for that position. Letter insertions were not scored. Table 2 indicates the percentage of letters correct according to their position, separately for each string length. As it can be seen, the pattern of results is quite clear: (1) all errors occurred on the left half of strings, and their rate increased progressively as the absolute distance from the centre of the string increased; (2) the point in a string to the left of which F.C. makes reading errors (i.e., neglect point, Ellis et al. [13]) shifted rightward for longer strings; (3) despite the fact that F.C. performed better on words than on non-words, the overall pattern of result was quite similar for both types of target.

### *3.2. Eye movements in reading*

A small percentage of saccades records (4% in normals and 7 % in patient F.C.) were contaminated by eye blinks and head movements. These trials were discarded and repeated later in a separate session.

*First saccade location.* Figure 1 shows the mean landing point of first saccades as a function of the length of words and non-words, separately for F.C. and control subjects. A two-factor ANOVA, with Lexicality (word, non-word), and String Length (6, 9, 10, 11 letters) as within-subject factors, was performed separately on controls' and patient's data. For normal subjects, the only significant effect was String Length $[F(3,9) = 22.8,\ P < 0.0002]$. The first fixation always fell to the left, close to the middle of the string (on average, 2.4 characters to the left), but as the string length increased from 6 to 11 letters there was a tendency for first saccade to land slightly nearer the beginning of the string.

In striking contrast with controls performance, in F.C. the first saccade was almost always directed to the right side of the stimulus (on average, 1.2 characters to the right). Furthermore, for F.C. but not for normals, the landing location of the first saccade was significantly modulated by the lexical status of the target. The landing location was displaced more towards the right for non-words than for words [1.53 vs 0.9 letters to the right; $F(1,6) = 32.1,\ P < 0.001$]. Also the effect related to String Length was significant $[F(3,18) = 8.4,\ P < 0.001]$. Landing location was displaced further rightward as string length increased.

*First saccade latency.* The mean latencies of first saccades made to word and non-word strings of various length are shown in Fig. 2. A two-factor ANOVA using Lexicality (word, non-word), and String Length (6, 9, 10, 11 letters) as within-subject factors was carried out on saccade latency data, separately for the control group and patient F.C.. For normal readers, there were slightly shorter saccade latencies for word than for non-word (203 vs 227 ms), although the effect of Lexicality failed to achieve significance $[F(1,3) = 2.9,\ P = 0.1]$. There was also a tendency for first saccade latency

Table 2
Percentage of letter correct as a function of letter position in word and non-word strings of different lengths for patient F.C.

| Length | | | | | | Word centre<br>X | | | | | |
|---|---|---|---|---|---|---|---|---|---|---|---|
| 6 | | | 57.1 | 85.7 | 100 | 100 | 100 | 100 | | | |
| 9 | | 42.9 | 42.9 | 71.4 | 85.7 | 100 | 100 | 100 | 100 | 100 | |
| 10 | | 14.3 | 14.3 | 28.6 | 85.7 | 85.7 | 100 | 100 | 100 | 100 | 100 |
| 11 | 14.3 | 14.3 | 28.6 | 28.6 | 42.9 | 57.1 | 100 | 100 | 100 | 100 | 100 |
| Length | | | | | | Non-word centre<br>X | | | | | |
| 6 | | | 14.3 | 28.6 | 100 | 100 | 100 | 100 | | | |
| 9 | | 0 | 0 | 14.3 | 28.6 | 85.7 | 100 | 100 | 100 | 100 | |
| 10 | | 0 | 0 | 0 | 14.3 | 28.6 | 57.1 | 71.4 | 100 | 100 | 100 |
| 11 | 0 | 0 | 0 | 0 | 0 | 42.9 | 85.7 | 100 | 100 | 100 | 100 |

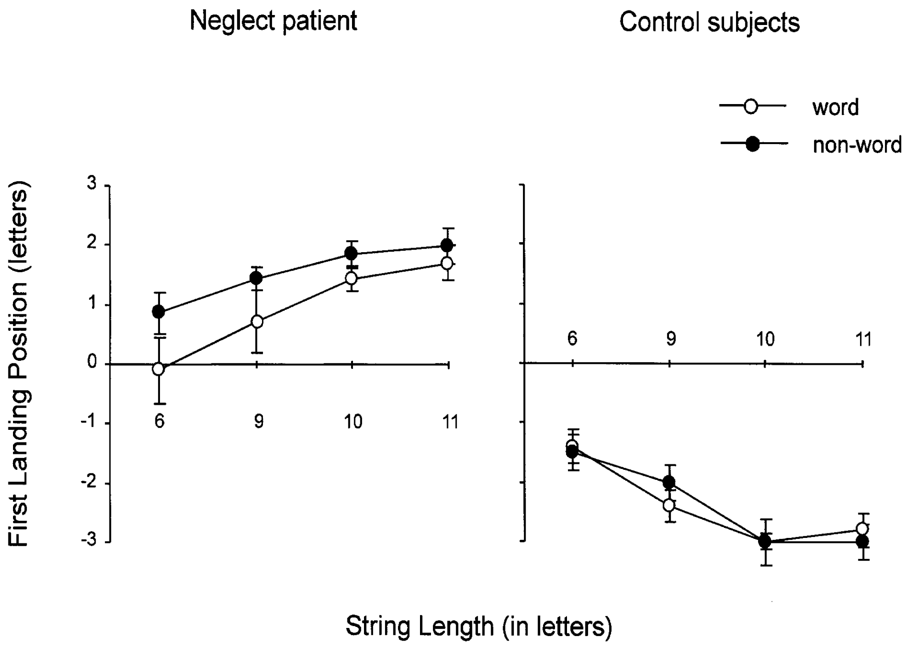

Fig. 1. Graphs plotted the mean first landing location on word and non-word strings as a function of string length (6, 9, 10, and 11 letters), for neglect patient F.C. (left graph) and control subjects (right graph). Landing location was expressed in terms of letter position in a string. Letters were numbered from the centre of the string to outwards, with right-sided letters coded as positive, and left-sided letters as negative. The number 0 on the y axis indicates the string centre.

to be shorter when short strings were presented, which was reflected in a marginally significant effect of String Length $[F(3,9) = 3.4,\ P = 0.06]$. The interaction was not significant ($F < 1$).

For F.C., the analysis of first saccade latency revealed a significant main effect of String Length $[F(3,18) = 6.7,\ P = 0.003]$. Saccades with longer latency were found following presentation of longer strings. In contrast, the main effect of Lexicality was not significant $[F(1,6) = 3.7,\ P = 0.1]$, although saccades made to words had shorter mean latency than those directed to non-words (219 and 234 ms, respectively).

*Distribution of fixation time.* The mean fixation time as a function of letter position for word and non-word stimuli is shown in Tables 3 and 4 for F.C. and control subjects, respectively. For the purpose of analysis, we also calculated the mean fixation duration for the left and right side of each stimulus (see Fig. 3). For odd-numbered strings, the fixation time on the central letter was equally divided between left and right side. Control subjects and patient data were analysed separately. In each case a three-factor ANOVA, with Lexicality (word, non-word), String Length (6, 9, 10, 11 letters), and Side (left, right) as within-subjects factors,

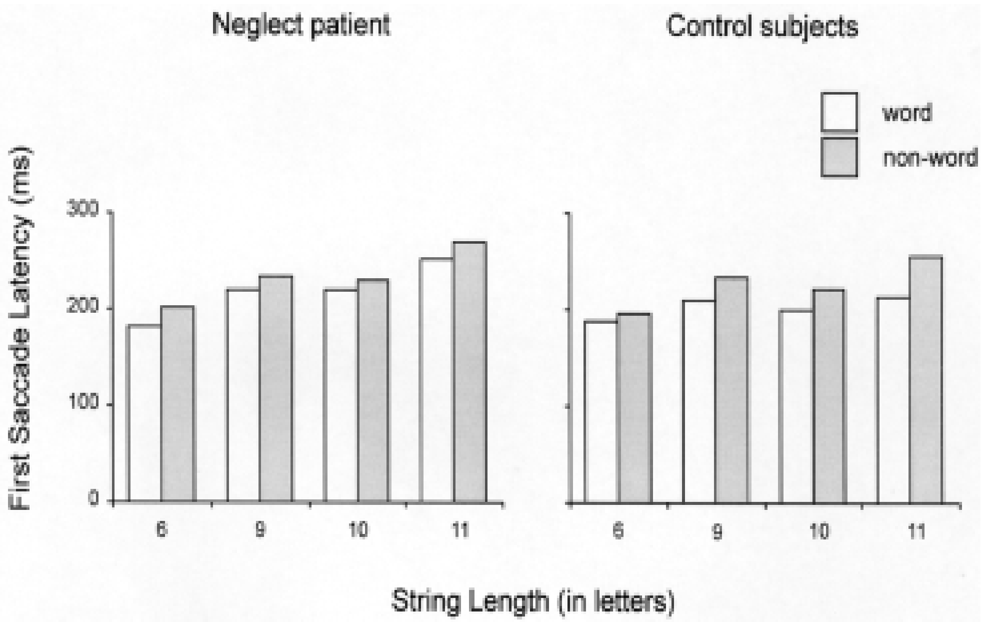

Fig. 2. Mean first saccade latencies obtained to word and non-word strings as a function of string length (6, 9, 10, and 11 letters), for neglect patient F.C. (left) and control subjects (right).

was conducted on the mean fixation time spent toward the left and right side of each string. Overall, the control group spent more time inspecting non-words than words $[F(1,3) = 39.2,\ P < 0.008]$. In addition, fixation times on the left were longer than those on the right [2370 vs 1506 ms; $F(1,3) = 54.3,\ P < 0.005$]. There was also a significant interaction between Lexicality and Side $[F(1,3) = 61.6,\ P < 0.004]$. Tukey post-hoc tests at $P < 0.05$ revealed that the difference in inspection duration between left and right side were significant for non-word targets (2786 vs 1339 ms), but not for word targets (1959 vs 1673 ms).

For F.C., analysis of data revealed no significant difference in inspection time between word and non-word targets [$F(1,6) = 0.64$, n.s.]. However, there was a significant main effect of String Length $[F(3,18) = 6.3,\ P < 0.004]$, with six-letter strings being inspected for less time than nine-, ten-, and eleven-letter strings. More importantly, there was a robust interaction between Lexicality and Side $[F(1,6) = 162.7,\ P < 0.0001]$. This interaction reflects the fact that, whereas for words F.C. spent more time on the left than on the right side of the stimulus (2061 vs 1213 ms; $p < 0.05$), the opposite effect occurred when non-word stimuli were displayed (927 vs 2205 ms; $p < 0.05$). There was also a significant interaction between String Length and Side $[F(3,18) = 7.4,\ P < 0.001]$. Paired comparisons with Tukey test showed that the difference in fixation durations between left and right side was confined to 11-letter strings ($p < 0.05$).

In addition to the data presented above, we also examined the relationship between F.C.'s fixation behaviour and his ability to report letters on the left (neglected) side of words. To this end, we carried out three separate regression analyses in which the number of left-sided letters correctly read for each word were regressed against a) the amount of fixation time spent toward the left side of the string, b) the amount of fixation time spent toward the right side, and c) the difference between the fixation time spent toward the left and right side of words. All regression analyses yielded significant linear relationships [a): $F(1,26) = 8.5,\ P < 0.007$; b): $F(1,26) = 14.1,\ P < 0.0009$; c): $F(1,26) = 30.5,\ P < 0.0001$]. However, the amount of variance accounted for by each regression was 24.7%, 35.2%, and 54.1%, respectively. In other words, we found that the factor that better predicted the probability of reporting left side letters was not the absolute time spent on the left or right side of words, but the difference in fixation duration between left and right side.

## 4. Discussion

This study was designed to document the eye movement behaviour during reading of F.C., a patient who

Table 3
Mean fixation time (in ms) as a function of letter position in word and non-word strings of different lengths for patient F.C.

| Length | Word centre X | | | | | | | | | | |
|---|---|---|---|---|---|---|---|---|---|---|---|
| 6 | 468 | 731 | 909 | 240 | 27 | 0 | | | | | |
| 9 | 100 | 312 | 451 | 890 | 936 | 434 | 113 | 24.9 | 0 | | |
| 10 | 30 | 199 | 391 | 446 | 103 | 814 | 399 | 276 | 27.3 | 0 | |
| 11 | 30 | 122 | 267 | 530 | 699 | 805 | 901 | 728 | 189 | 52.3 | 0 |
| Length | Non-word centre X | | | | | | | | | | |
| 6 | 51.7 | 298 | 612 | 662 | 422 | 300 | | | | | |
| 9 | 0 | 34 | 78.3 | 512 | 1189 | 867 | 465 | 124 | 0 | | |
| 10 | 0 | 0 | 14.3 | 201 | 673 | 1000 | 667 | 712 | 215 | 63.4 | |
| 11 | 0 | 0 | 30.9 | 33.1 | 249 | 772 | 1106 | 868 | 388 | 163 | 30.9 |

Table 4
Mean fixation time (in ms) as a function of letter position in word and non-word strings of different lengths for control subjects

| Length | Word centre X | | | | | | | | | | |
|---|---|---|---|---|---|---|---|---|---|---|---|
| 6 | 214 | 490 | 879 | 841 | 323 | 155 | | | | | |
| 9 | 22.8 | 286 | 551 | 640 | 986 | 605 | 414 | 249 | 55.5 | | |
| 1 | 11 | 309 | 508 | 543 | 692 | 727 | 475 | 399 | 121 | 30.3 | |
| 11 | 59.3 | 347 | 428 | 482 | 459 | 811 | 672 | 392 | 257 | 80.3 | 0 |
| Length | Non-word centre X | | | | | | | | | | |
| 6 | 528 | 804 | 902 | 1141 | 383 | 36.3 | | | | | |
| 9 | 277 | 592 | 713 | 720 | 758 | 624 | 464 | 233 | 0 | | |
| 10 | 248 | 696 | 652 | 634 | 720 | 590 | 375 | 136 | 69.8 | 0 | |
| 11 | 187 | 506 | 669 | 811 | 796 | 619 | 267 | 232 | 32.3 | 0 | 9 |

presented a reliable left neglect dyslexia following a right hemisphere damage. As F.C. showed a word superiority effect [1,4,6,28,31,32,50] (i.e., he was much more impaired when naming non-word than word strings), the primary goal of the study was to examine whether the lexical status of stimuli also influenced the patient's eye movement performance. We analysed the pattern of eye movements during reading, with the assumption that it represents a moment-to-moment index of the allocation of attention through visual space [9, 18,26,27,54]. As indicated in the Introduction, the fact the neglect dyslexia affects more non-word than word reading has received a variety of explanations which differ, among other things, with regard to the spatial distribution of attention. One such account suggests that neglect subjects are more likely to guess the initial letters of words than to guess those of non-words by virtue of the orthographic regularities of written language, and postulates an invariance of spatial attention across different lexical stimuli [43]. An alternative interpretation emphasizes the facilitatory effect of pre-existing lexical knowledge on the identification of the leftmost and less visible letters if they are part of words. Activation of a lexical representation by the input would, in turn, trigger a shift of attention toward the critical left side of the word in order to encompass the entire length of the stimulus [6]. Thus, the interaction between attention and lexical factors remains controversial, and we used F.C.'s eye movement data to gain new insight into this issue.

A second focus of interest of this article was to examine whether neglect dyslexia emerges as a primary deficit in making leftward saccades. Indeed, despite a number of eye movement studies in visual neglect [5, 16,17,22,59], very little is known about eye movement patterns during inspection of individual words and non-words in neglect dyslexia.

Before considering the neglect patient, we briefly summarise the eye movement data from the normal subjects. The group of normals showed a spatial asymmetry in their visual exploration of the letter strings: (1) They systematically directed their first saccade to the left, about halfway between the beginning and the centre of the string.[1] As string length increased, this landing location tended to move a bit farther to the

[1] This location of initial fixation has been labelled preferred viewleft, nearer the beginning of the string; (2) on average, normal subjects spent an equal amount of time viewing the left and right side of words. In contrast, for non-words they spent more time fixating the left side than the right side. Moreover, this asymmetry in oculomotor exploration increased as the string increased in length. The above findings are compatible with prior research demonstrating that, when normal subjects inspect words, they spent more time fixating letters which occupy a fairly central position of the string [42,46,47]. In the case of difficult or rare words (or non-words, as in our case), which may require a slow, serial mechanism of grapheme-to-phoneme translation, majorities of fixations tend to fall more to the left, in a region which enables a more efficient identification of each individual letter of the string [42,47].

As to the neglect patient, the experiments indicate that F.C.'s reading performance was susceptible to the lexical status of the items as evident by his better naming with words than with non-words. Furthermore, he produced more neglect errors as the number of letters in a string increased. These results are reflected in F.C.'s pattern of visual exploration during reading which appeared very different to that observed in normals. First, we will consider the landing location of the first saccade on word and non-word strings, then discuss the distribution of fixation time across stimuli, and examine the relationship between fixations and visual awareness.

### 4.1. First saccade location

In striking contrast to the performance of normal subjects, in the majority of trials the patient's first saccades were directed to the right side of the string, irrespective of the length and lexical status of the stimulus. This abnormal pattern conforms well with the eye movement data reported previously for neglect patients [5,16,17,22,59], and is a clear indication of F.C.'s chronic orienting bias to the ipsilesional side. However, results showed that both length and lexical attributes of the string influenced the landing location of the first saccade. This location fell progressively more rightward as stimulus length increased and, most important, differed significantly between words and non-words. Indeed, we found the mean landing location on non-words was about 1 character positions to the right with respect to the first fixation location on words.

ing location. Landing at this position allows to see clearly initial letters and to minimise recognition time [47].

These results clearly demonstrate that lexical representations have been activated some time prior to the decision to make the first saccade (which on average occurred 230 ms after stimulus presentation), and support the position that the lexical properties of the stimulus affect eye movements at very early stage of word recognition processing [28,31,32]. The present findings are in complete agreement with recent event-related potential (ERP) studies of normal reading which have demonstrated effects of lexicality (i.e., words vs non-words) on the early components of the ERP waveform (P1 and N1), that is approximately 100–150 ms post stimulus onset [53]. Similar estimates of the amount of time required to distinguish between words and non-words have also been reported in a number of behavioural studies in normal perceivers [39,45].

One question that needs to be addressed is why the first fixation position on non-words suffered a stronger rightward bias (i.e., neglect) than the landing location on words, despite the fact that both types of stimuli provide similar low-level inputs (letters) to the attentional-oculomotor mechanisms. One hypothesis [6,28,56,57] maintains that a word can be treated as a single (lexical) object since it has a unitised code stored in memory. As a consequence, it can be selected as a whole and results less subjected to a spatial attention disorder. Conversely, non-words may be regarded as a spatial layout of multiple concurrent perceptual items, which need to be processed individually and thus compete one against the other for focal attention. Indeed, the presence of multiple perceptual units (vs a single visual item) in a display has been shown to lead to a greater ipsilesional bias (and severe contralesional neglect) in many other perceptual tasks [29,30]. The hypothesis that neglect is reduced when visual stimuli can be treated as a single perceptual object is also supported by other eye movement studies [23,53]. These reported evidence of patients who are not (or only mildly) impaired at making saccades to the left side of individual objects, despite being impaired at making leftward saccades when viewing multiple visual stimuli. For instance, Rizzo and Hurtig [53] described patients who failed at making saccades to the left side of a multi-object scene, but not into the left side of a face stimulus.

In addition to high-level lexical properties of the items, F.C.'s first landing location was also sensitive to low-level perceptual features of the stimulus, as his performance was clearly affected by the length of the string. These results suggest that perceptual (visual) and lexical (cognitive) analysis of the string largely overlap in time, so that both types of operations may affect the eye-movement/attentional mechanism from early phases of word recognition. This point argues against the existence of separate and completely autonomous perceptual and lexical processes, as proposed by some models of normal word identification [15]. Conversely, our finding is in line with the interactive activation model of word recognition of McClelland and Rumelhart [36,37] which postulates that visual processing occurs at different levels at the same time, with activation at low- and high-level that simultaneously exert control over attentional allocation and behaviour. Moreover, these results appear highly compatible with a recent computational model of eye movement control in reading, E-Z Reader [48], which assumes that both visual and linguistic variables affect eye-movement behaviour during reading.

### 4.2. *Distribution of fixation time*

We examine now the distribution of fixation time across stimuli. While on average F.C. spent more time fixating the left side of words, with non-words he always tended to fixate more on the right side of the stimulus. Thus, results revealed that the critical difference between control subjects and neglect patient F.C. in the distribution of fixation durations across strings was largely confined to the exploration of non-words, whereas for words the distribution of inspection time was quite similar.

In addition, the magnitude of such oculomotor (attentional) rightward bias was affected by the spatial extent of stimuli. As the number of letters in a string increased from 6 to 11 letters, F.C.'s tendency to spend more time fixating the ipsilesional (right) side of the stimulus also increased.

The first implication of these results is that the distribution of attention (as indexed by eye movements) in neglect dyslexia is not only a function of spatial location of stimuli, but it is also deeply modulated by the nature of the objects presented in the visual field [6,19]. F.C. was able to make saccades to the contralesional side and spent more time fixating there when the stimulus was a word. However, he appeared to restrict his eye fixations to the right side when the stimulus was a non-word. Thus, for F.C. the shifting of attention within letter string was determined by lexical factors. With non-words strings, which do not activate the lexicon, F.C.' s eye fixations seem determined mainly by the initial position where gaze (and attention) is oriented. By contrast, with words, which strongly activate the lexicon, the patient reorient attention to the leftmost end of the string following the initial orienting response to the right side.

To sum up, this study provides direct evidence that reading of non-words is accompanied by a greater rightward bias in the allocation of attention (and, as a consequence, more profound neglect dyslexia) than reading of words. Similar data have been reported in previous works with neglect subjects [6,28,57]. For instance, Brunn and Farah [6], who used a secondary tasks to assess the distribution of attention during reading, have also found evidence of greater contralesional attention for words than non-words in neglect dyslexia. In one of these tasks, subjects had to mark the centre of a line that was presented directly underneath word and non-word strings. They reported that line bisection was much more symmetrical with words than non-words.

Overall our results do not support the view that the relative sparing of words compared to non-word is independent of attention and based upon an inferential process, as suggested by Patterson and Wilson [43]. By contrast, our data provide direct evidence of multiple lexical influences on eye movements (and attention) in neglect dyslexia, which probably reflect different levels of lexical activation reached during reading. It seems plausible to assume that lexical access is not an all-or-none process (which is complete within the first saccade latency), but a process that occurs gradually (see McClelland and Rumelhart [36,37] on this point), and influences visual attention at multiple stages during stimulus identification. On this view, initial lexical activation has an immediate effect over the first overt orienting response, as we have reported here. Then, as stimulus encoding progresses, the magnitude of lexical activation increases and may therefore induce a reorienting of attention to the left in order to encompass the entire area subtended by the word. The observation of lexically driven refixations to the left side of words is in keeping with McConkie's assumption [38] that a saccadic eye movement is initiated whenever the attentional system is seeking visual information from a retinal area which is not readily identified. Thus, when a word is presented to a neglect dyslexic patient, initial lexical activation enhances (although not completely) the visibility of left side letters. This partial registration, in turn, may provide a signal to move the eye (and the focus of attention) toward left side letters in order to acquire there additional perceptual information. By contrast, when non-words are displayed, information from the left side of the string is not improved by the internal lexical knowledge and can not trigger any reallocation of attention to its location.

It is important to point out that word strings need not to be fully identified to influence the reallocation of attention. As stated in the Introduction, Ládavas and her colleagues [31,32] have recently showed a preserved lexical access in neglect dyslexic patients notwithstanding their poor performance in reading words and nonwords aloud. Thus, lexical activation may elicit a shift of attention and gaze toward the left side for further processing; however, such refixations may have only a minor effect on reading due to the patient' s spatial selection impairment toward the contralesional side.

### 4.3. *Fixation and visual awareness*

We now turn to an important and intriguing aspect of the present findings, that is the surprisingly large amount of time that F.C. spent fixating on the "neglected" left side of words, while denying awareness of visual information thereof. Indeed, for 6-, 9-, and 10-letter words, the patient spent more time on the left than on the right side of the word, and yet failed to name 19%, 47%, and 67% of letters on the left, respectively. For example, in one trial he fixated for over 1700 ms on the left side of the word "AMBULANZA" (Italian for "ambulance") and only reported right sided letters ("LANZA"). A similar evidence has been recently provided by Walker et al. [60] in a patient with left visual neglect who gazed (for over 2 seconds), at the left side of a chimaeric face, but named only the half face on the right. The presence of leftward eye movements without a corresponding stimulus detection has been reported in another study by Làdavas et al. [33], suggesting again a functional dissociation of the mechanisms subserving attentional and gaze orienting.

The results of the present study, as well as the previous ones [33,60], are clear-cut: they establish that F.C.'s neglect cannot simply be explained in terms of a failure to fixate or make saccades toward the neglected side of the stimulus. Rather, the evidence suggests that F.C.' pattern of eye movements is the consequence of a more serious disorder of visuo-spatial representation. These findings also rule out an account of F.C.'s neglect dyslexia in terms of a selective visual field defect, which might have been undetected by standard campimetry, since the patient failed to become aware of the left letters even when they fell entirely in his right (intact) field of vision. Finally, the fact that left side letters can be disregarded or poorly reported even when fixated confirms that neglect dyslexia arises from a major deficit of selection, rather than from an elementary perceptual or motor impairment. The data from F.C. are compatible with recent studies demonstrating that the time course of visual selection is abnormally extended in neglect (and extinction) [11,21]. For instance, Husain et al. [21] found in neglect subjects that a letter that must be identified continues to occupy attentional capacity for more than 1400 ms. The fact that F.C. did not report left side letters of words despite of their prolonged inspection, is thus consistent with recent neuropsychological models that portray visual neglect as a deficit disrupting the allocation of attention in space, as well as in time [20].

We also looked at the relationship between F.C.'s pattern of eye movements and his ability to successfully report left side letters of words. We found that a longer fixation duration on the left-side of words was associated with a higher percentage of reported letters on that side. However, the factor that appeared to better predict F.C.'s reading performance towards the neglected side was the differential fixation time between left and right side of the word. That is, crucial to awareness and overt report of left-sided letters was not the absolute fixation time on the left side, but its value relative to the right side. We think that such a finding may have direct implications for the nature of the attentional deficit underlying F.C.'s neglect dyslexia. It suggests that conceptions of neglect based on impaired functioning confined to one hemispace – either in terms of an orienting deficit to contralesional items [19] or an impaired disengagement from ipsilesional stimuli [44] – are not completely adequate characterisations of this spatial disorder. More plausible explanations are those that see neglect (and extinction) not as an absolute impairment to deal with one side of space, but more as the result of a competitive imbalance between orienting biases directed to opposite side of space [8,10,25,34, 61]. This imbalance appears strongly modulated by the attributes of the displayed stimulus, as we have shown here for F.C.

In his recent "integrated competition" model of visual selection, Duncan [12] specifically argues that attention is a state in which the different properties of a selected stimulus become available together for control of behaviour. This suggestion nicely fits with the present results which provide evidence that, in neglect dyslexia, the physical features of the selected stimulus (i.e., length) and its more abstract properties (i.e., lexical form) influence together the attentional/oculomotor bias against contralesional letters from very initial stages of visual word recognition.

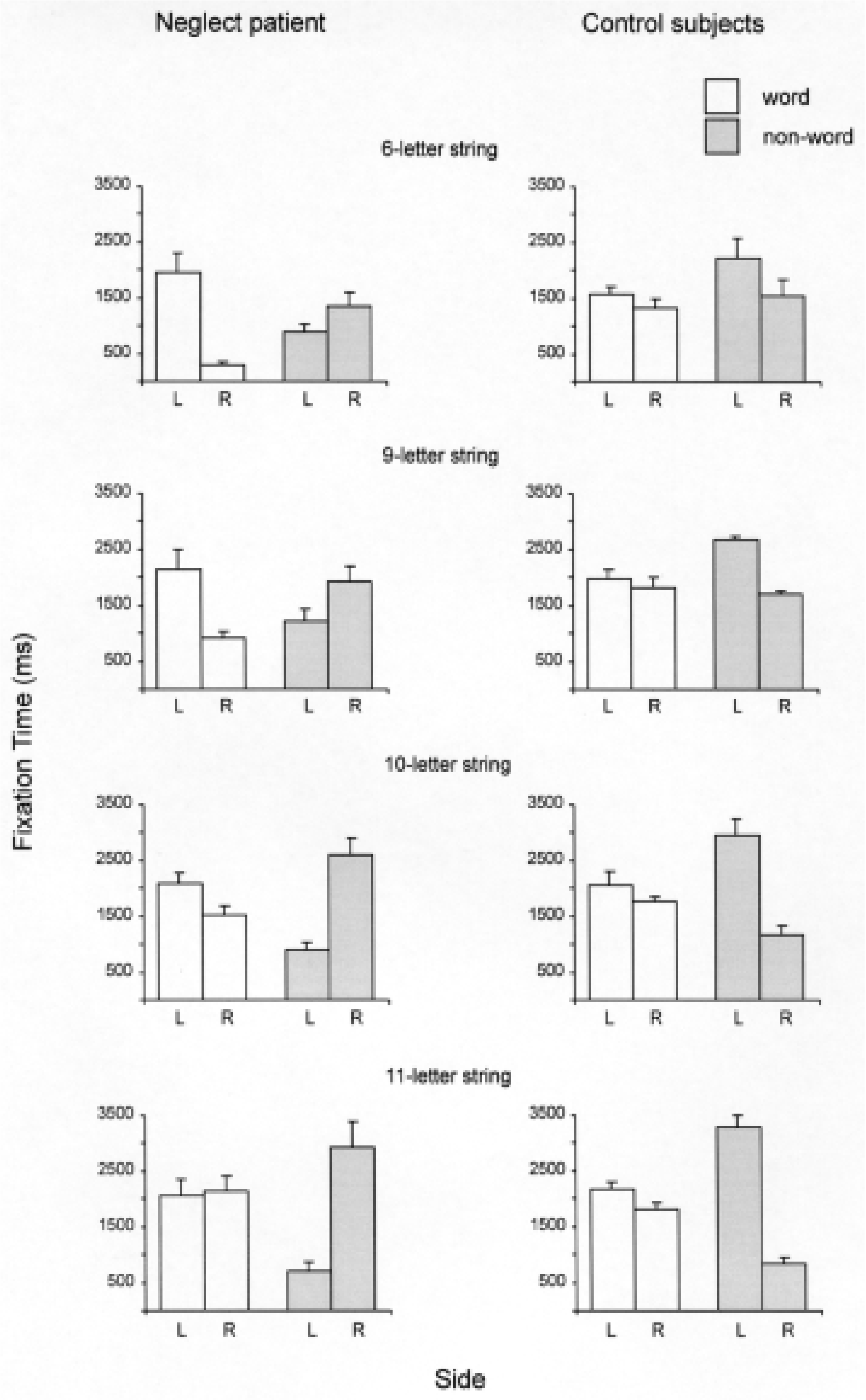

Fig. 3. Graphs plotted the mean fixation time to the left (L) and right (R) side of word and non-word strings of 6, 9, 10, and 11 letters in length, for neglect patient F.C. (left graphs) and control subjects (right graphs).
